# Percutaneous Preoperative Biliary Drainage for Resectable Perihilar Cholangiocarcinoma: No Association with Survival and No Increase in Seeding Metastases

**DOI:** 10.1245/s10434-015-4676-z

**Published:** 2015-06-30

**Authors:** Jimme K. Wiggers, Bas Groot Koerkamp, Robert J. Coelen, Alexandre Doussot, Susan van Dieren, Erik A. Rauws, Mark A. Schattner, Krijn P. van Lienden, Karen T. Brown, Marc G. Besselink, Geert van Tienhoven, Peter J. Allen, Olivier R. Busch, Michael I. D’Angelica, Ronald P. DeMatteo, Dirk J. Gouma, T. Peter Kingham, Joanne Verheij, William R. Jarnagin, Thomas M. van Gulik

**Affiliations:** Department of Surgery, Academic Medical Center Amsterdam, Amsterdam, The Netherlands; Department of Surgery, Erasmus Medical Center Rotterdam, Rotterdam, The Netherlands; Department of Hepatobiliary and Pancreatic Surgery, Memorial Sloan Kettering Cancer Center, New York, NY USA; Clinical Research Unit, Academic Medical Center Amsterdam, Amsterdam, The Netherlands; Department of Gastroenterology, Academic Medical Center Amsterdam, Amsterdam, The Netherlands; Department of Gastroenterology, Memorial Sloan Kettering Cancer Center, New York, NY USA; Department of Interventional Radiology, Academic Medical Center Amsterdam, Amsterdam, The Netherlands; Department of Interventional Radiology, Memorial Sloan Kettering Cancer Center, New York, NY USA; Department of Radiation Oncology, Academic Medical Center Amsterdam, Amsterdam, The Netherlands; Department of Pathology, Academic Medical Center Amsterdam, Amsterdam, The Netherlands

## Abstract

**Background:**

Endoscopic biliary drainage (EBD) and percutaneous transhepatic biliary drainage (PTBD) are both used to resolve jaundice before surgery for perihilar cholangiocarcinoma (PHC). PTBD has been associated with seeding metastases. The aim of this study was to compare overall survival (OS) and the incidence of initial seeding metastases that potentially influence survival in patients with preoperative PTBD versus EBD.

**Methods:**

Between 1991 and 2012, a total of 278 patients underwent preoperative biliary drainage and resection of PHC at 2 institutions in the Netherlands and the United States. Of these, 33 patients were excluded for postoperative mortality. Among the 245 included patients, 88 patients who underwent preoperative PTBD (with or without previous EBD) were compared to 157 patients who underwent EBD only. Survival analysis was done with Kaplan–Meier and Cox regression with propensity score adjustment.

**Results:**

Unadjusted median OS was comparable between the PTBD group (35 months) and EBD-only group (41 months; *P* = 0.26). After adjustment for propensity score, OS between the PTBD group and EBD-only group was similar (hazard ratio, 1.05; 95 % confidence interval, 0.74–1.49; *P* = 0.80). Seeding metastases in the laparotomy scar occurred as initial recurrence in 7 patients, including 3 patients (3.4 %) in the PTBD group and 4 patients (2.7 %) in the EBD-only group (*P* = 0.71). No patient had an initial recurrence in percutaneous catheter tracts.

**Conclusions:**

The present study found no effect of PTBD on survival compared to patients with EBD and no increase in seeding metastases that developed as initial recurrence. These data suggest that PTBD can safely be used in preoperative management of PHC.

Patients diagnosed with perihilar cholangiocarcinoma (PHC) typically present with obstructive jaundice, which impairs liver function and is a risk factor for mortality after hepatobiliary surgery.^1^ Preoperative biliary drainage can resolve jaundice before surgery and may help reduce perioperative morbidity in patients submitted to en bloc partial hepatectomy.^2^ In Western centers, patients are preoperatively treated with endoscopic biliary drainage (EBD), percutaneous transhepatic biliary drainage (PTBD), or both.

PTBD has been the preferred preoperative drainage method in Asian centers for decades, with favorable perioperative morbidity and mortality rates, but recent studies have focused on seeding metastases after preoperative PTBD and resection.^3^–^5^ These seeding metastases presumably result from exfoliated tumor cells in bile that drains along the percutaneous catheter. The incidence of catheter tract recurrences in those studies ranged from 2 to 5 %, and the incidence of laparotomy scar recurrences was 1.3 %.^6^–^9^ A low rate of seeding metastases has been reported since the early years of preoperative PTBD, but it is only recently that this low rate has been used to advocate for an exclusively endobiliary strategy.^10^,^11^ On the basis of the above data, many Eastern authors recently suggested that preoperative PTBD should be avoided and that endoscopic nasobiliary drainage should be preferred.^12^–^14^ From an oncologic perspective, however, only recurrences that affect overall survival (OS) are clinically relevant recurrences. It remains unclear if the reported seeding metastases were solitary recurrences, if they coincided with other recurrences, or if they developed after recurrent metastatic disease. Moreover, none of the above studies have assessed the effect of preoperative PTBD on OS.

The present study was designed to assess OS after resection of PHC in patients with preoperative PTBD compared to patients with preoperative EBD. Additionally, we assessed the incidence of seeding metastases developing as initial recurrence after resection because we assumed that these initial recurrences would potentially influence OS. The broader objective was to establish the role that PTBD should have in preoperative management of PHC: either as a drainage method that can safely be used or only as a salvage procedure when other methods have failed.

## Methods

### Study Population

Consecutive patients who underwent a resection with curative intent for PHC were identified from prospectively maintained databases at the Academic Medical Center (AMC) in Amsterdam, the Netherlands, and the Memorial Sloan Kettering Cancer Center (MSKCC), New York. PHC was defined according to the 7th edition of the American Joint Committee on Cancer staging manual.^15^ Patients were included from 1991 to 2012 if they had undergone preoperative biliary drainage before resection of PHC using extrahepatic bile duct resection and reconstruction with or without concomitant liver resection. Exclusion criteria were R2 resection, repeat resection after initial resection at another hospital, and 90 day postoperative mortality. Additional data were collected through retrospective chart review. The institutional review board at both institutions approved this study.

Patient selection for resection and preoperative management was similar between the 2 centers, as described previously.^16^ Biliary drainage was initiated in either a regional center before referral, or after referral to AMC or MSKCC. Patients were treated with initial EBD or initial PTBD according to the treating physician’s preference. Additional preoperative PTBD was performed when biliary decompression was inadequate after EBD or if EBD was associated with complications, such as cholangitis. The PTBD group in this study included patients treated with initial PTBD and patients treated with additional PTBD after inadequate EBD. The control group consisted of patients treated with preoperative EBD without previous or subsequent PTBD.

All patients in the AMC in Amsterdam were routinely treated with a preoperative low-dose irradiation protocol (3 × 3.5 Gy in the 3 days before the resection) with the aim of preventing seeding metastases.^17^ Entry sites of percutaneous drain tracts in the abdomen were not routinely excised after resection.

### Follow-up After Resection

All patients were followed with CT imaging at 3 and 6 months after resection to detect early recurrence. Thereafter, patients at AMC were followed with clinical assessments until 5 years after resection; imaging was performed when indicated by rising tumor markers, symptoms, or findings at physical examination. At MSKCC, follow-up included CT or MRI imaging every 4 to 6 months. Pathologic confirmation of recurrences was often obtained, but it was not required if imaging unambiguously demonstrated recurrent disease in patients who were unfit to undergo further treatment. Suspect lesions at the laparotomy scar or in the prior PTBD drainage tract were always confirmed with a biopsy.

### Outcomes

The primary end point in this study was OS, measured from the date of surgery to the date of death. Patients alive at follow-up were censored at the date of last contact before April 1, 2014. We used propensity score method rather than traditional multivariable analysis because this method is considered superior in reducing confounding and bias, especially when analyzing relatively small observational data sets.^18^,^19^

Secondary end points were directed toward the incidence of seeding metastases after resection. Analysis of recurrences focused on the pattern of initial recurrences based on the assumption that prognosis was unlikely to be affected by seeding metastases that arose after recurrence at another site. Seeding metastases were defined as recurrences either in the percutaneous catheter tract (i.e., any recurrence along the catheter tract from skin to the intrahepatic bile duct) or in the laparotomy scar (i.e., any recurrence in the abdominal wall at the laparotomy scar).^6^,^8^ In addition, the incidence of peritoneal recurrences (i.e., intra-abdominal recurrence in the peritoneum or ascites with malignant cells) was assessed, although these recurrences were not necessarily regarded as seeding metastases.

### Statistical Analyses

Our method of propensity score adjustment was straightforward. First, we estimated propensity scores for the probability of PTBD assignment on the basis of all observed baseline characteristics. Second, we analyzed OS with a Cox proportional hazards model including 2 variables: drainage method (PTBD vs. EBD only) and propensity score (continuous variable). This model adjusts the survival analysis conditional on the propensity score. Thus, the model calculates the effect of PTBD compared to EBD only given that the propensity scores (i.e., the observed baseline characteristics) are hold equal.

In more detail, we calculated propensity scores using multivariable logistic regression with preoperative PTBD as the outcome of interest and with adjustment for observed baseline characteristics, including demographics, comorbidities, total bilirubin level at referral, the level of bile duct involvement (Bismuth class), preoperative imaging variables (Blumgart T stage), cholangitis, extended hepatectomy, and treating center. Three baseline characteristics had missing data, including bilirubin level at presentation (27.8 % missing), Blumgart T stage (6.9 % missing), and preoperative cholangitis (5.3 % missing). To avoid bias, multiple imputation with 10 imputed data sets was performed for these missing data before estimation of the propensity scores, using a regression model that included all baseline characteristics. To evaluate residual bias after adjustment for propensity score, logistic regressions with drainage method as outcome were performed for each of the baseline characteristics with and without adjustment for propensity scores. We then estimated OS using the Kaplan–Meier method, and compared the groups with the log-rank test in univariable analysis. Finally, a multivariable Cox proportional hazards model was used to compare OS between the PTBD and EBD-only groups after adjustment for the propensity score as a continuous variable. To assess the proportional hazards assumption, we inspected the hazard ratio plots and found no violation.

Analysis of secondary end points was performed by *χ*^2^ tests, and 95 % confidence intervals (CIs) were determined using the standard deviation of the mean. The type of liver resection and pathologic characteristics were also compared by *χ*^2^ tests. All analyses were performed in SPSS v22 (IBM, Armonk, NY).

## Results

### Patients

A total of 344 consecutive patients underwent resection of PHC during the study period, of whom 66 (19.2 %) were excluded because they had not undergone preoperative biliary drainage. Of the remaining 278 patients, 33 (11.9 %) were excluded for 90 day postoperative mortality: 3 (8.1 %) of 37 patients treated with preoperative PTBD; 17 (9.8 %) of 147 patients treated with preoperative EBD; and 13 (19.4 %) of 67 patients treated with both. As a result, 245 patients were included, comprising 128 treated at MSKCC and 117 at AMC. Patient characteristics were not different between MSKCC and AMC, except for older age at MSKCC (mean age 65 vs. 61, respectively). The policy to use preoperative PTBD was different between the centers: PTBD was more often used in MSKCC than in AMC (43.8 vs. 27.4 %, respectively; *P* = 0.008).

The PTBD group consisted of 88 patients (36 %) who were treated with preoperative PTBD, including 54 patients who underwent PTBD after inadequate EBD. Patients in the PTBD group had undergone a median of 2 preoperative PTBD procedures (range 1–5). The median time between the first PTBD drainage procedure and surgery was 38 days (range 3–262); 17 patients (19.3 %) had a percutaneous catheter in situ more than 60 days. The EBD-only group (i.e., the control group) consisted of 157 patients (64 %) who were treated with preoperative EBD without PTBD. The distribution of patients between the PTBD and EBD-only groups was equal throughout the study period. The percentage of PTBD procedures between 1991 and 1996 was 32.3 %; 1997 and 2001, 37.0 %; 2002 to 2006, 32.7 %; and 2007 to 2012, 38.2 % (*P* = 0.88).

Baseline characteristics of the study groups are presented in Table 1. Nearly all variables were different between groups, indicating severe bias at baseline. The mean ± standard deviation propensity scores for patients in the PTBD and EBD-only groups were 0.53 ± 0.24 and 0.27 ± 0.19, respectively, with an area under the curve of 0.79. Only minimal differences in baseline characteristics remained after adjustment for propensity score, as indicated by the adjusted *P* values in Table 1. Type of resection and pathologic characteristics of both study groups are shown in Table 2.

**Table 1:** Baseline characteristics

Variable	PTBD (*n* = 88)	EBD only (*n* = 157)	*P*	
			Imputed	Imputed adjusted for propensity score
Male	53 (60.2)	100 (63.7)	0.59	0.94
Age, *y*, median (IQR)	61 (16)	65 (13)	0.01	0.99
Comorbidity, Charlson score ,^27^ median (IQR)	0 (1)	0 (1)	0.92	0.81
Total bilirubin at referral, μmol/L, median (IQR)	135 (231)	39 (67)	0.02	0.92
Bismuth class on imaging			0.001	0.95
Type 1	8 (9.1)	41 (26.1)		
Type 2	11 (12.5)	23 (14.6)		
Type 3a	30 (34.1)	44 (28.0)		
Type 3b	18 (20.5)	28 (17.8)		
Type 4	19 (21.6)	16 (10.2)		
Left or right hepatic duct	2 (2.3)	5 (3.2)		
Blumgart T stage on imaging			0.004	0.96
1	38 (43.7)	86 (61.0)		
2	28 (32.2)	40 (28.4)		
3	21 (24.1)	15 (10.6)		
Preoperative cholangitis	25 (29.4)	13 (8.8)	<0.001	0.89
Extended hepatectomy	43 (48.9)	44 (28.0)	0.001	0.99
Treating center			0.008	0.98
MSKCC	56 (43.8)	72 (56.3)		
AMC	32 (27.4)	85 (72.6)		

Data are presented as *n* (%) unless otherwise specified. Logistic regressions with drainage method as outcome (PTBD or EBD only) were performed for each of the baseline variables to evaluate residual bias after adjustment for propensity scores

*PTBD* percutaneous transhepatic biliary drainage, *EBD* endoscopic biliary drainage, *IQR* interquartile range, *MSKCC* Memorial Sloan Kettering Cancer Center, *AMC* Academic Medical Center

**Table 2:** Type of liver resection and pathologic characteristics

Characteristic	PTBD (*n* = 88)	EBD only (*n* = 157)	*P*
Preoperative cytology assessment, *n* (%)			0.40
Positive or suspicious	49 (55.7)	93 (59.2)	
Negative	18 (20.4)	39 (24.8)	
Not performed	21 (23.9)	25 (15.9)	
Type of liver resection, *n* (%)			0.003
Extrahepatic bile duct resection only	10 (11.4)	38 (24.2)	
Segment 4/5 wedge resection	3 (3.4)	17 (10.8)	
Mesohepatectomy	2 (2.3)	0 (0)	
Left hemihepatectomy	22 (25.0)	34 (21.7)	
Left extended hemihepatectomy	8 (9.1)	12 (7.6)	
Right hemihepatectomy	5 (5.7)	17 (10.8)	
Right extended hemihepatectomy	38 (43.2)	39 (24.8)	
Resection including caudate lobe^a^	46 (61.3)	63 (61.8)	0.08
Resection specimen, *n* (%)			
T3 or T4 tumor (AJCC 7th edition)	34 (38.6)	31 (19.7)	0.002
R1 resection	21 (23.9)	49 (31.2)	0.24
Moderate/poor differentiation	22 (25.0)	33 (21.0)	0.52
Perineural invasion	70 (79.5)	108 (68.8)	0.08
Resected lymph nodes			
Total lymph node count, median (range)	3 (1–22)	4 (1–20)	0.15
N1 lymph node metastasis, *n* (%)	30 (34.1)	35 (22.3)	0.05
Mean lymph node ratio (positive/negative)	0.14 (1/7)	0.09 (1/11)	0.03

*PTBD* percutaneous transhepatic biliary drainage, *EBD* endoscopic biliary drainage, *AJCC* American Joint Committee on Cancer

^a^ Percentage of caudate resections only concerns patients who underwent meso- or hemihepatectomy

### Overall Survival

**Fig. 1:**
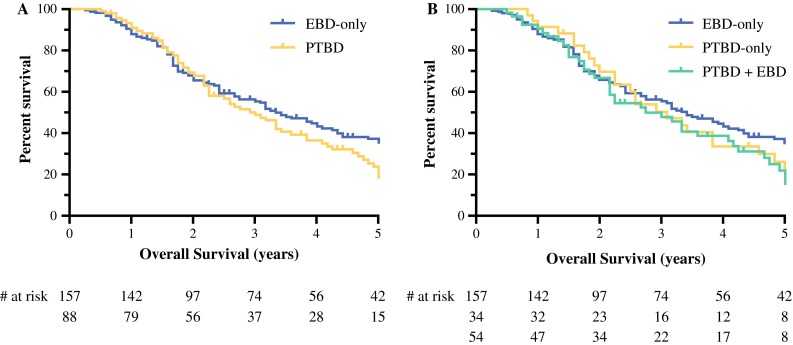
Unadjusted Kaplan–Meier survival plots. **a** Patients in PTBD and EBD-only groups had comparable survival (*P* = 0.26). **b** Stratifying patients in PTBD group between those who underwent PTBD only and those who underwent PTBD plus EBD did not reveal difference (*P* = 0.45)

**Fig. 2:**
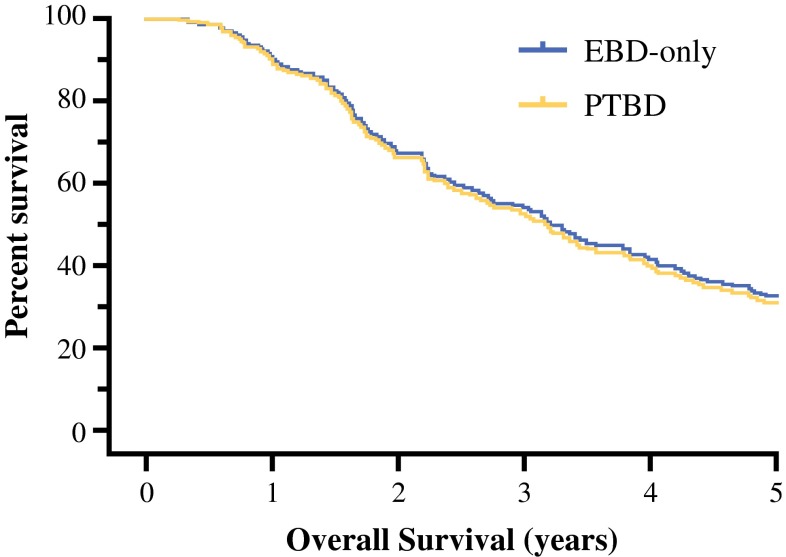
Survival plot after adjustment for propensity score in Cox regression analysis showing similar OS in PTBD and EBD-only groups (*P* = 0.80)

Among the 245 included patients, 173 patients (71 %) died during follow-up. The median OS was 38 months (95 % CI 32–44), and 5-year survival was 32 %. Median follow-up among survivors was 52 months (range 6–251 months). The unadjusted OS was comparable between the PTBD group (36 months) and the EBD-only group (41 months; *P* = 0.25; Fig. 1a). Stratifying patients in the PTBD group between those who underwent EBD plus PTBD and those who underwent PTBD only did not reveal a difference when these 2 groups were compared to patients who underwent EBD only (*P* = 0.44; Fig. 1b). After using propensity score adjustment to account for potential confounders, OS between the PTBD group and EBD-only group was similar (adjusted hazard ratio, 1.05; 95 % CI 0.74–1.49; *P* = 0.80; Fig. 2).

### Seeding Metastases Developing as Initial Recurrence

A total of 87 patients in the PTBD group and 147 patients in the EBD-only group were available for recurrence analysis (1 patient missing in the PTBD group and 10 patients missing in the EBD-only group; total 4 %). Seeding metastases occurred as initial recurrence in 3 (3.4 %) of 87 patients in the PTBD group (95 % CI 0–7.3), and in 4 (2.7 %) of 147 patients in the EBD-only group (95 % CI 0–5.3; *P* = 0.71). Among the total 7 patients who developed a seeding metastasis, 3 had developed a concurrent local recurrence. Time to diagnosis of the seeding metastases was 8, 13, 14, 17, 20, 21, and 66 months (median, 17 months), and OS in these 7 patients was 13, 30, 20, 21, 21, 27, and 142 months (median, 21 months), respectively. All 7 seeding metastases developing as initial recurrence were abdominal wall recurrences at the site of the laparotomy scar. No initial recurrences were observed in a percutaneous catheter tract. The incidence of seeding metastases was not significantly different between both centers: 5 (3.9 %) of 128 patients at MSKCC (95 % CI 0.5–7.3) and 2 (1.9 %) of 106 patients at AMC (95 % CI 0–4.5; *P* = 0.46).

### Peritoneal Recurrences

Initial peritoneal recurrences were observed in 32 (13.7 %) of 234 patients with available recurrence status (95 % CI 9.2–18.1). These included 11 (12.6 %) of 87 patients in the PTBD group and 21 (14.3 %) of 147 patients in the EBD-only group, which was not significantly different between groups (*P* = 0.85). Concomitant peritoneal recurrence was observed in only 1 of 7 patients with a seeding metastasis as initial recurrence.

## Discussion

Biliary drainage has become an important component of the preoperative preparation of patients with PHC, as multiple studies have shown that it decreases postoperative liver failure and mortality.^2^ Nonetheless, several controversies have evolved. Eastern centers reported catheter tract recurrences after preoperative PTBD and resection of PHC and promoted the use of alternative drainage methods. In the present study, however, we showed that preoperative PTBD is not associated with survival after resection of PHC compared to patients who underwent preoperative endoscopic drainage. Moreover, PTBD was not associated with an increase in seeding metastases developing as initial recurrence that would potentially affect survival.

One previous study has assessed survival after preoperative PTBD: in a study of 141 patients with resected PHC, Hirano et al. found a median OS of 31 months after preoperative PTBD compared to 59 months after preoperative EBD.^20^ The authors attributed this difference to an increase in peritoneal metastases after preoperative PTBD. However, the PTBD group in that study had more advanced disease as evidenced by more patients with Bismuth type 4 tumors (30 vs. 14 %), more perioperative blood transfusions (31 vs. 9 %), and more frequent hepatic artery resections (22 vs. 9 %). Although a survival difference was confirmed in multivariable analysis, the statistical model in that study may have been at risk to a false-positive finding due to overfitting because it was adjusted for 9 other covariates. Moreover, statistical criteria, like adjusting for all significant variables from univariable analysis, as used in the study by Hirano et al., are considered insufficient to characterize confounding or selection bias.^21^

Catheter tract recurrences have been reported in a range of 2 to 5 % after preoperative PTBD, but our study found no catheter tract recurrence developing as the initial recurrence after resection.^6^–^9^ This discrepancy may be partly explained by the behavior of seeding metastases. Apparently, catheter tract recurrences, if they occur at all, have a tendency to grow slowly and not to manifest before other recurrences have been diagnosed. Alternatively, differences in management and patient selection between centers in the present study and Eastern centers could explain the discrepancy. The duration of PTBD has been identified as a risk factor for catheter tract recurrences: preoperative PTBD longer than 60 days was associated with an increased risk in the study by Takahashi et al., and more than 25 % of the patients in that study reached the cutoff, compared to only 19 % in the present study.^6^ It is uncertain whether preoperative low-dose radiotherapy, which was standard treatment in the AMC and not in MSKCC, has prevented catheter tract recurrences or other seeding metastases. There was no difference in the incidence of seeding metastases between the 2 study centers, so the current data does not support routine use of preoperative radiotherapy.

Normally, PHC spreads to the liver and through lymph nodes to the abdomen or extra-abdominal sites.^22^ In analogy to previous studies, we named recurrences in the laparotomy scar or in the percutaneous catheter tract “seeding metastases” because these recurrences show a deviating pattern from normally observed recurrences and are likely the result of tumor seeding. Nonetheless, clear evidence for a role of seeding tumor cells has not been demonstrated in these kinds of recurrences. Alternatively, local inflammation after surgical trauma might naturally attract circulating tumor cells.^23^ To a larger extent, peritoneal recurrences are doubtfully the result of tumor seeding. Although some peritoneal metastases may be caused by perioperative tumor seeding and could thus be preventable, most peritoneal metastases will reflect extensive disseminated disease. In the present study, preoperative PTBD did not increase the incidence of peritoneal recurrences.

This retrospective study has several limitations. The sample size was likely insufficient to definitively exclude an adverse effect of preoperative PTBD on OS after resection of PHC. Although no statistically significant difference was found when comparing unadjusted OS and propensity-adjusted OS, the 95 % CI of the propensity-adjusted hazard ratio was still relatively wide. However, not a single catheter tract recurrence was found during follow-up of initial recurrences, and the incidence of initial abdominal wall recurrences was also similar between the PTBD and EBD-only groups.

The analysis of seeding metastases as initial recurrence requires 3 comments. First, follow-up was not standardized, so it is possible that some initial seeding metastases were missed after patients were lost to follow-up. Second, we only recorded initial recurrences, and we may have missed seeding metastases that occurred after initial diagnosis of recurrent disease. This follow-up approach may not provide the true incidence of seeding metastases, but it is based on clinical meaningfulness: management or OS is unlikely to be affected by seeding metastases if they occur late in the course of the disease, after the initial diagnosis of recurrence. Third, the present study included only patients who underwent a potentially curative resection, so patients with inoperable disease due to (extra)hepatic or N2 lymph node metastases were excluded. On the basis of these data, we cannot be sure whether some patients had seeding metastases at the time of surgery. Nonetheless, in our experience with management of PHC, we have never observed any seeding metastases during exploratory laparotomy.

Regarding management of PHC, the use of preoperative drainage before smaller liver resections (e.g., left hemihepatectomy) may not be necessary because of the large liver remnant. Moreover, preoperative drainage could even be harmful in these patients, as a recent study showed that preoperative drainage might increase perioperative morbidity due to infection-related complications.^24^ In the present study, preoperative drainage was often used before small liver resections because many patients present to our centers with drains already in place or with badly placed drains that are associated with infection and require revision. Of note, 48 extrahepatic bile duct resections without liver resection were performed for Bismuth type 1 or 2 tumors during the early years of the study cohort. Since approximately 2000, a liver resection is part of a potentially curative resection for PHC, particularly for Bismuth type 2.

Preoperative PTBD for PHC is currently being used in most Western surgical specialty centers when EBD fails to obtain adequate preoperative biliary drainage. Some centers even prefer to use preoperative PTBD as primary drainage method instead of EBD. There are many reasons to use PTBD: it has been associated with fewer preoperative complications than EBD; percutaneous catheters provide direct access to bile ducts perioperatively; and percutaneous catheters can be used as stents to protect hepaticojejunostomies from leaking postoperatively.^25^ No definitive data are currently available for any of the suggested advantages, but a randomized controlled trial is being conducted to assess differences in perioperative complications between EBD and PTBD.^26^

In conclusion, these data suggest that PTBD can safely be used in preoperative management of PHC. The present study found no effect of PTBD on survival compared to patients who underwent preoperative EBD and no increase in seeding metastases that develop as initial recurrence. The decision to use preoperative PTBD should not be influenced by concerns about catheter tract recurrences; they are very rare, and they probably do not affect OS.
